# Editorial: Distributed and embodied cognition in scientific contexts

**DOI:** 10.3389/fpsyg.2023.1207262

**Published:** 2023-05-30

**Authors:** Majid D. Beni, Ahti-Veikko Pietarinen, Mirko Farina

**Affiliations:** ^1^Department of Philosophy, Middle East Technical University, Ankara, Türkiye; ^2^Department of Religion and Philosophy Hong Kong Baptist University, Kowloon, Hong Kong SAR, China; ^3^Faculty of Humanities and Social Science, Innopolis University, Innopolis, Russia

**Keywords:** social cognition, distributed cognition, embodied cognition, extended cognition, dynamic systems

Three decades have passed since the distributed cognition programme began to modify the landscape of the cognitive sciences (Hutchins, 1995, 2000; Kirsh, 2006). Distributed cognition indicates that cognitive systems are individuated not by specific borders or boundaries (such as skin or skull) but as distributed in socio-cultural environments that situate individuals, artifacts, and patterns of their scaffolded collaborations across multiple scales. Among others, the distributed cognition programme has inspired extended, embodied, and enactive views on cognition (Clark and Chalmers, 1998; Clark, 2005; Kirsh, 2010; Farina and Lavazza, 2022a,b), and with those and other complementary and reciprocal approaches, it has been seen as one of the mainstays of the contemporary cognitive sciences.

The contributions included in the present Research Topic improve upon our understanding of distributed cognition in two significant ways. On the one hand, they unpack the theoretical implications of this paradigm in the domains of epistemology and philosophy of science, the topic that to date has remained somewhat less researched in the literature. On the other hand, some of the contributions presented in this Research Topic advance preceding research by aligning it with breakthroughs in computational embodied/extended neuroscience. Among them are the theory and application of the free energy principle (Friston, 2011; Clark, 2016), which have previously resulted in some fairly sophisticated accounts of social cognition (Friston and Frith, 2015), with implications for the social aspects of science (Nersessian and Chandrasekharan, 2009; Beni, 2021) and scientific methodology (Beni and Pietarinen, 2021; Pietarinen and Beni, 2021). The present Research Topic signposts some recent developments in distributed cognition across the multiplicity of scientific contexts.

Hipólito and van Es's “*Enactive-dynamic social cognition and active inference*” draws on the formal framework of dynamical systems theory (DST) to explain the origins of socio-cognitive novelty. The negative part of Hipólito and van Es's proposal addresses what is regarded by them as a confusion about active inference, which is sometimes introduced as a mix of the classic Theory of Mind (ToM) and enactivism. The positive part of their contribution offers a genuinely enactivist view on active inference as a modeling tool that (when cast in the framework of DST) explicates social understanding as the generalized synchronization observed in the natural and life sciences.

Pritchard's “*Socially extended scientific knowledge*” charts out the general organization of social cognition and allocates distributed scientific knowledge in that structure, in tandem with developing a virtue-theoretic account of the extension of *cognitive agency* that is characteristically found in collaborative scientific enterprises. This proposal provides a viable way of demarcating genuine cases of bona fide technologically extended scientific knowledge from cases in which technology only facilitates scientific knowledge (in the former case, technology is sufficiently integrated into the agent's cognitive practices to be a proper part of the extended cognition process, whereas in the latter case, technology merely facilitates cognition of the unextended cognitive process without being integrated into the process).

Gillett et al., building up and expanding on Kaplan's (2012) seminal work on the mutual manipulability criterion (MM), defend the legitimacy of using mechanistic strategies to demarcate the bounds of cognition. In other words, by drawing from the literature on constitutive mechanistic explanations in the philosophy of science, the authors of this paper develop a new and improved version of MM, which they claim can be instrumental in assessing putative cases of extended cognition and hence in pushing forward the debate about the locus of cognition.

Di Rienzo's paper proposes bridging the gap between scientific knowledge and its practical applications (known in the literature as the “knowledge-to-action” gap) by situating the analysis of distributed cognition in the context of clinical practices, which often involve non-linear interactions and synergies between materials, socio-cultural environments, and people skillfully engaging with them.

The distributed cognition programme may benefit from a formal account of the dynamic interplay between the agent and its environment. Indeed, Miller et al.'s contribution, “*Resilience and active inference*”, offers a free energy-based account of the “fit” between an agent and their physiological, physical, or cultural niche. To achieve this goal, they offer a conceptual analysis of *resilience* in terms of inertia onto high-precision beliefs, resilience as elasticity onto relaxation back to characteristic (i.e., attracting) states, and resilience as plasticity onto functional redundancy and structural degeneracy.

In “Examining ecological and enactivist approaches to modeling disability”, Jurgens raises an issue with the commitment of enactivist approaches to an individualist methodology. After arguing that the problem haunts both the theoretical and practical aspects of enactivist models, Jurgens suggests that the enactivist approach could be revamped by adopting elements from the neurodiversity paradigm and Robert Chapman's ecological–functional approach.

The stated goal of this Research Topic has been to investigate and critically evaluate the relevance, importance, and significance of the current status of distributed cognition in a variety of scientific contexts. We hope our readers are equipped with some valuable insights into this fascinating and lively Research Topic.

## Author contributions

All authors listed have made a substantial, direct, and intellectual contribution to the work and approved it for publication.
